# Editorial: Mechanisms Underlying Diabetes-Associated Damage in Target Tissues

**DOI:** 10.3389/fphar.2022.915806

**Published:** 2022-05-17

**Authors:** Beatriz Suarez-Alvarez, Raúl R. Rodrigues Díez

**Affiliations:** ^1^ Translational Immunology Laboratory, Instituto de Investigación Sanitaria del Principado de Asturias (ISPA), Oviedo, Spain; ^2^ Molecular and Cellular Biology in Renal and Vascular Pathology, IIS-Fundación Jiménez Díaz-Universidad Autónoma Madrid, Madrid, Spain

**Keywords:** diabetes, pancreatic β cell, kidney, liver, skin

It is estimated that close to 463 million adults are currently living with diabetes and a rise to 700 million is predicted by 2045. There are multiple complications associated with diabetes, and both type 1 and type 2 diabetes (T1D and T2D), constitute a major source of morbidity and mortality inducing macrovascular (coronary artery disease, peripheral arterial disease, and stroke) and microvascular (diabetic nephropathy, neuropathy, and retinopathy) complications, being T2D the most prevalent (around 90%). Apart from these Research Topic, the importance of T2D in the development and progression of other pathologies, such as Non-Alcoholic Fatty Liver Disease (NAFLD), has also been reported. Current management of diabetes includes new anti-hyperglycemic medications such as the sodium-glucose cotransporter-2 (SGLT-2) inhibitors, glucagon like peptide-1 receptor agonist (GLP-1 RA), endothelin A receptor blockade and anti-inflammatory drugs, having proved to be effective in preventing the progression to diabetes complications as demonstrated in preclinical and clinical studies. However, to further unravel the underlying mechanisms of diabetes-associated damage in target tissues new studies are necessary.

In this Research Topic of Frontiers in Pharmacology, we expose new evidence and therapeutical approaches of the pathophysiological mechanism associated to diabetes-related diseases.

Nowadays, the incidence of diabetic nephropathy (DN) is alarmingly growing in the world due to the increase of T2D linked to metabolic syndrome, obesity and dyslipidaemia. Despite hyperglycaemia control managing, these patients progress toward end-stage renal disease (ESRD) partially explained by the hyperglycemic “*metabolic memory*” concept, and the underlying mechanisms such as inflammation and fibrosis. According to these principles, Opazos-Rios et al. analysed the microRNA expression profile in the kidney of an experimental model of T2D and obesity. The authors showed a renal damage in those animals similar to that reported in patients with advanced DN, as well as an increase of miRNAs mainly associated to inflammatory and immune processes (Th1 response). Other involved pathways regulated by miRNAs, such as necroptosis, adipogenesis or epithelial-to-mesenchymal transition reveal the complexity of this disease, highlighting the need for a multifactorial therapeutic approach. A similar context is present in the diabetic retinopathy where hypoxia, inflammation, oxidative stress or vascular permeability contribute jointly to the progression of the disease. Regarding this issue, Howell et al. demonstrated that an ERK5 inhibitor, XMD8-92, is able to decrease the diabetes-mediated retinal inflammation, vascular endothelial growth factor (VEGF) production, and oxidative stress in streptozotocin (STZ)-induced diabetic mice. The effect of this small inhibitor, that can cross the blood-retina-barrier, is not only mediated by the blockage of ERK5 downstream pathways, but also by damaging the activity of the BRD4 epigenetic modulator.

Hepatopathies had been widely documented to be also associated to diabetes, mainly the NAFLD as a common comorbidity of T2D. However, although the epidemiological data are quite limited, individuals with T1D might also be at an increased risk of developing NAFLD and glycogenic hepatopathy (GlyH). In these terms, Mertens et al. reviewed the factors contributing to the development of these pathologies in patients with T1D and proposed an interesting guide of recommendations for the diagnosis and the differentiation of these two chronic liver diseases. First studies revealed that NAFLD might contribute to the increase in the risk of cardiorenal morbidity and mortality associated to T1D, close to that reported in T2D. The authors describe the pathophysiologic mechanisms mediated by dyslipidaemia, oxidative stress, immune activation, glycogen metabolism, intrahepatic fat synthesis, composition of nutrients or pancreatic hormones leading to diabetes-induced liver damage. Appropriately, the recently coined term “metabolic dysfunction-associated fatty liver disease” (MALFD) as alternative to NALFD is discussed in the context of T1D. Despite GlyH is infrequent and associated with poorly controlled T1D, it could be underdiagnosed in those patients because liver biopsy is the only method for its identification. Thus, this overview paves the avenue for new lines of research to properly identify these hepatopathies and their consequences in long-term outcome of T1D patients.

Another common T2D associated complication is the development of foot ulcers that, in some cases, may result in minor amputations or even in the loss of an extremity or dead. The delay in the wound healing process observed in T2D patients participates in the development and progression of these ulcers. Endothelial precursor cells (EPCs) play an important role in wound healing process and there is evidence that EPC number and function are impaired in diabetic patients compared to healthy individuals. In this Research Topic Wu et al. demonstrated that oral uptake of nicotinamide riboside (NR), a new form of vitamin B3, enhanced EPC function to promote diabetic wound healing in diabetic mice, proposing that NR supplementation might be a promising strategy to prevent the progression of diabetic complications in humans.

A wide range of molecules has been described to participate in the generation and progression of pancreatic β cell disfunction that leads to T2D. Here, Jing et al. demonstrated a novel signalling mechanism induced by the serotonergic agonist specific for the 5-hydroxytryptamine 2c receptor (5-HT2CR), lorcaserin, that can induce detrimental effects in the function of pancreatic β cell by inhibiting glucose-stimulated insulin secretion (GSIS) and calcium influx. Although lorcaserin was approved by Food and Drug Administration (FDA) in 2012 for chronic weight management, in 2020 the same organization requested its withdrawal from the market due to increasing diagnosis in cancer-related cases after lorcaserin treatment. Then, this article provides T2D development risk as a new aspect to be considered in future studies testing this drug. On the contrary, Zhang et al. contributed to unravel the mechanism implicated in the potential beneficial effects attributed to the Sphingosine 1-phosphate (S1P) regulating glucose metabolism and, therefore, T2D development. In their study, authors demonstrated that S1P enhanced GSIS in pancreatic β cells as well as promoted their survival by inhibiting the voltage-dependent potassium (Kv) channels. In addition, authors showed that Kv channels blockade by S1P was induced by the phospholipase C (PLC)/protein kinase C (PKC) signalling pathway activation.

PPARγ, a nuclear receptor, is another key molecule in the glucose and lipid metabolism. Accordingly, PPARγ agonists are currently being used for the treatment of dyslipidaemia and T2D, although some questions remain already under debate. The differences between PPARγ partial or full agonists had been previously evaluated to reduce the side effects, such as heart problems, bone loss, weight gain or fluid retention. In their article, Cao et al. examined the effect two PPARγ agonists, rosiglitazone (full agonist) and CMHX008 (partial agonist) on the cardiac hypertrophy in obese mice models and compared them to the findings in T2D patients under treatment with rosiglitazone. Authors showed that obesity is associated with a higher expression of PPARγ2 in cardiomyocytes leading to the cardiac hypertrophy. Moreover, they reported that rosiglitazone induces an increase of hypertrophy-related genes and early enhancement of the myocardial damage when treating obese mice and T2D patients respectively. By contrast, CMHX008 has a similar protective effect in the cardiac dysfunction but without modifying cardiomyocytes’ structure. Thus, this study confirms the use of novel PPARγ partial agonist as the ideal antidiabetic agent since it improves insulin sensitivity without compromising the cardiac function. Following this line of research, Vales-Villamarin et al. showed the correlation of the Pro12Ala polymorphism of the PPARγ2 gene in children with the susceptibility to develop T2D. They found that leptin seems to impact on the association between the PPARγ2 Pro12Ala and BMI, presumably because this polymorphism leads to a decreased binding of PPARγ2 to the gene encoding leptin, downregulating its expression.

Altogether, the results compiled in this Research Topic expand the knowledge about the mechanisms implicated in T1D and T2D development as well as the associated complications in the kidney, liver, retina and skin, proposing new potential targets and therapeutic strategies that should be further corroborated.

